# The long-term effects of dapagliflozin in chronic kidney disease: a time-to-event analysis

**DOI:** 10.1093/ndt/gfae106

**Published:** 2024-05-10

**Authors:** Phil McEwan, Peter D Gabb, Jason A Davis, Juan Jose Garcia Sanchez, C David Sjöström, Salvatore Barone, Pavlos Kashioulis, Mario Ouwens, Syd Cassimaty, Ricardo Correa-Rotter, Peter Rossing, David C Wheeler, Hiddo J L Heerspink

**Affiliations:** Health Economics and Outcomes Research Ltd, Cardiff, UK; Health Economics and Outcomes Research Ltd, Cardiff, UK; Health Economics and Outcomes Research Ltd, Cardiff, UK; Health Economic and Payer Evidence, AstraZeneca, Cambridge, UK; Late-Stage Development, Cardiovascular, Renal, and Metabolism, AstraZeneca, Gothenburg, Sweden; Global Medical Affairs, AstraZeneca, Gaithersburg, USA; Late-Stage Development, Cardiovascular, Renal, and Metabolism, AstraZeneca, Gothenburg, Sweden; Medical & Payer Evidence Statistics, AstraZeneca, Gothenburg, Sweden; Global Commercial Integration, AstraZeneca, Cambridge, UK; Department of Nephrology and Mineral Metabolism, National Medical Science and Nutrition Institute Salvador Zubiran, Mexico City, Mexico; Steno Diabetes Center Copenhagen, Herlev, Denmark; Department of Clinical Medicine, University of Copenhagen, Copenhagen, Denmark; Department of Renal Medicine, University College London, London, UK; Department of Clinical Pharmacy and Pharmacology, University of Groningen, Groningen, The Netherlands; The George Institute for Global Health, Sydney, Australia

**Keywords:** chronic kidney disease, chronic renal failure, diabetic kidney disease, mortality, SGLT2 inhibitor

## Abstract

**Background:**

Chronic kidney disease (CKD) presents a significant clinical and economic burden to healthcare systems worldwide, which increases considerably with progression towards kidney failure. The Dapagliflozin and Prevention of Adverse Outcomes in Chronic Kidney Disease (DAPA-CKD) trial demonstrated that patients with or without type 2 diabetes who were treated with dapagliflozin experienced slower progression of CKD versus those receiving placebo. Understanding the effect of long-term treatment with dapagliflozin on the timing of kidney failure beyond trial follow-up can assist informed decision-making by healthcare providers and patients. The study objective was therefore to extrapolate the outcome-based clinical benefits of treatment with dapagliflozin in patients with CKD via a time-to-event analysis using trial data.

**Methods:**

Patient-level data from the DAPA-CKD trial were used to parameterize a closed cohort-level partitioned survival model that predicted time-to-event for key trial endpoints (kidney failure, all-cause mortality, sustained decline in kidney function and hospitalization for heart failure). Data were pooled with a subpopulation of the Dapagliflozin Effect on Cardiovascular Events – Thrombolysis in Myocardial Infarction 58 (DECLARE-TIMI 58) trial to create a combined CKD population spanning a range of CKD stages; a parallel survival analysis was conducted in this population.

**Results:**

In the DAPA-CKD and pooled CKD populations, treatment with dapagliflozin delayed time to first event for kidney failure, all-cause mortality, sustained decline in kidney function and hospitalization for heart failure. Attenuation of CKD progression was predicted to slow the time to kidney failure by 6.6 years [dapagliflozin: 25.2, 95% confidence interval (CI) 19.0–31.5; standard therapy: 18.5, 95% CI 14.7–23.4] in the DAPA-CKD population. A similar result was observed in the pooled CKD population with an estimated delay of 6.3 years (dapagliflozin: 36.0, 95% CI 31.9–38.3; standard therapy: 29.6, 95% CI 25.5–34.7).

**Conclusion:**

Treatment with dapagliflozin over a lifetime time horizon may considerably delay the mean time to adverse clinical outcomes for patients who would go on to experience them, including those at modest risk of progression.

## Graphical Abstract

**Graphical Abstract:**
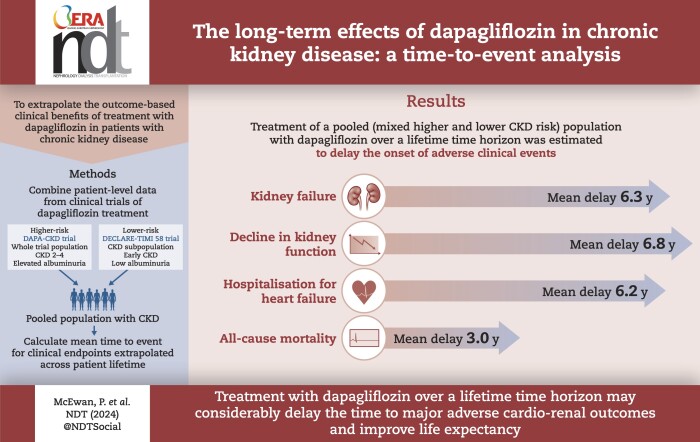

KEY LEARNING POINTS **What was known:**

- In the Dapagliflozin and Prevention of Adverse Outcomes in Chronic Kidney Disease (DAPA-CKD) trial, treatment of patients with or without type 2 diabetes (T2D) with dapagliflozin was associated with significant reductions to the incidence of CKD progression, hospitalization for heart failure and death from any cause, leading to its approval for use in patients with CKD regardless of T2D status.
- The Dapagliflozin Effect on Cardiovascular Events – Thrombolysis in Myocardial Infarction 58 (DECLARE-TIMI 58) trial estimated the effect of dapagliflozin in patients with T2D with or at risk of atherosclerotic disease. In this population, dapagliflozin significantly reduced the incidence of hospitalization for heart failure and the renal composite endpoint.
- Randomized, controlled clinical trials are typically conducted over a follow-up period of up to 4 years, which may not fully demonstrate the full clinical value in the treatment of chronic, progressive diseases.

**This study adds:**

- Using patient-level data from the DAPA-CKD trial, we estimate that long-term treatment with dapagliflozin may considerably delay the time to adverse cardio-renal outcomes and death from any cause. Notably, we estimated delayed CKD progression through treatment with dapagliflozin to slow the time to kidney failure by 6.6 years.
- By forming a pooled CKD population with the addition of a subpopulation of the DECLARE-TIMI 58 trial with CKD at baseline, we estimated this larger CKD cohort, including patients with less advanced CKD than the DAPA-CKD cohort, to experience comparable delays in kidney-related outcomes and hospitalization for heart failure.

**Potential impact:**

- Further characterization of treatment benefits beyond what is demonstrated in clinical trials can assist informed decision making by healthcare providers and patients.
- Our analysis suggests that long-term treatment with dapagliflozin has the potential to substantively delay the time to adverse outcomes including kidney failure, which could have beneficial consequences for healthcare service delivery.
- A delay in reaching kidney failure could substantially improve the health-related quality of life of patients and additional life years could be spent without the need for kidney replacement therapies.

## INTRODUCTION

The global prevalence of chronic kidney disease (CKD) is estimated at 11%–14% [1, 2], with over 850 million people affected worldwide [3]. Patients’ CKD may progress to end-stage kidney disease, or kidney failure, necessitating kidney replacement therapy in the form of dialysis or kidney transplantation as life-extending therapies. Dialysis is costly to the healthcare service in terms of resources and healthcare service delivery [4], burdensome to patients’ health-related quality of life [5] and typically requires frequent visits to a healthcare facility. Therefore, an unmet need exists for therapies to slow the progression of kidney function decline, prolong life expectancy and reduce downstream adverse cardio-renal effects.

An emerging option for patients with CKD secondary to several kidney pathologies is dapagliflozin, a sodium-glucose co-transporter 2 (SGLT2) inhibitor and an established therapy for patients with type 2 diabetes (T2D) that has demonstrated significant reductions in cardiovascular- and kidney-related outcomes [6–9]. Dapagliflozin is an example of this drug class that has been studied across multiple randomized controlled trials. Kidney-protective effects (distinct from glucose-lowering ones) were observed in the Dapagliflozin Effect on Cardiovascular Events – Thrombolysis in Myocardial Infarction 58 (DECLARE-TIMI 58) trial involving patients with T2D with or at risk of atherosclerotic disease [9] and the subsequent Dapagliflozin and Prevention of Adverse Outcomes in Chronic Kidney Disease (DAPA-CKD) trial evaluated efficacy and safety, focused on patients with CKD, with or without T2D [6]. Treatment with dapagliflozin led to significant reductions in the composite and individual component endpoints for CKD progression, risk of hospitalization for heart failure and death from any cause [10].

A common limitation of randomized, controlled clinical trials is that they have a typical follow-up period of up to 4 years, yet patients are often treated over a much longer period in clinical practice. Therefore, despite their large size, the DECLARE-TIMI 58 and DAPA-CKD trials may not fully demonstrate the clinical value of dapagliflozin in treating a chronic, progressive disease. Furthermore, clinical trials are powered to demonstrate risk reduction only over the time horizon of the trial; extrapolating the treatment effect beyond the follow-up period to estimate delayed time to adverse outcomes can provide evidence to support informed decision-making by patients and clinicians, and resource planning for healthcare providers.

The objectives of this study were to quantify the long-term benefits of dapagliflozin extrapolated beyond the time horizon of typical clinical trials, and to determine whether kidney/cardiovascular benefits are observed in early, intermediate and advanced stages of CKD using patient level data from the DAPA-CKD and DECLARE-TIMI 58 trials.

## MATERIALS AND METHODS

### Analysis populations

The analysis considered two populations: the DAPA-CKD population and a pooled CKD population, comprising both the DAPA-CKD population and a subset of the DECLARE-TIMI 58 trial population that met the criteria for a CKD diagnosis at baseline. The analysed trials were subject to ethical review and trial participants provided written informed consent as recorded in the associated publications [6, 9].

**Figure 1:**
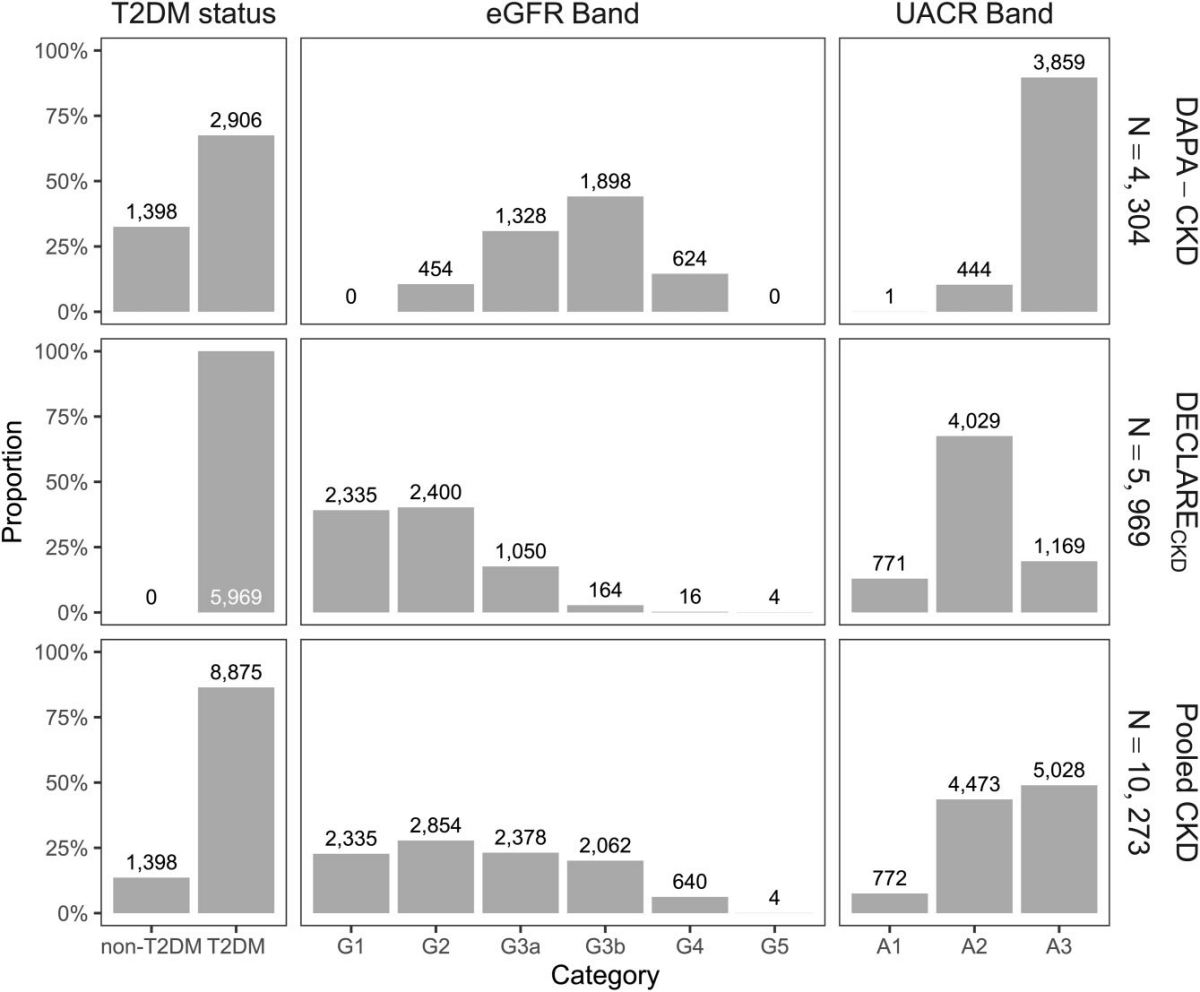
Patient baseline disease characteristics, stratified by T2D status, and eGFR/UACR bands as defined by KDIGO guidelines [14]. Bars represent proportions while superimposed numbers correspond to patient counts in each category. T2DM, type 2 diabetes mellitus.

The DAPA-CKD population reflected the eligibility criteria for the DAPA-CKD trial, namely patients with or without T2D, with estimated glomerular filtration rate (eGFR) 25–75 mL/min/1.73 m^2^ and urine albumin-to-creatinine ratio (UACR) 200–5000 mg/g; details of the DAPA-CKD study design and patient characteristics have previously been published [6, 11]. The DECLARE-TIMI 58 trial did not include CKD diagnosis as part of its inclusion or exclusion criteria, but baseline trial data were used to inform the risk of CKD according to Kidney Disease: Improving Global Outcomes (KDIGO) criteria (eGFR <60 mL/min/1.73 m^2^ or UACR >30 mg/g) [12]. Filtering by these criteria yielded a subset of the trial population with CKD, defined as DECLARE_CKD_ (*n* = 5969) [9, 13]. The DECLARE_CKD_ population consisted of patients with T2D who typically had higher eGFR levels at baseline and lower levels of albuminuria than patients in the DAPA-CKD trial (Fig. 1), thus representing patients with less advanced CKD relative to the DAPA-CKD population. The summary baseline characteristics for the analysis populations are detailed in Table 1.

**Table 1:** Summary patient characteristics of analysis populations.

		
Variable	DAPA-CKD (*N* = 4304)	Pooled CKD (*N* = 10 273)
		
Age, years	61.8 (12.1)	63.3 (9.6)
Female	1425 (33.1)	3365 (32.8)
Race		
Asian	1467 (34.1)	2367 (23.0)
Black	191 (4.4)	411 (4.0)
Other	356 (8.3)	581 (5.7)
White	2290 (53.2)	6914 (67.3)
Baseline body mass index, kg/m²	29.5 (6.2)	31.3 (6.3)
Current smoker	584 (13.6)	1482 (14.4)
Baseline systolic blood pressure, mmHg	137.1 (17.4)	137.3 (16.7)
Baseline diastolic blood pressure, mmHg	77.5 (10.5)	77.8 (9.8)
Haemoglobin, g/dL	12.8 (1.8)	13.4 (1.7)
Baseline serum potassium, mq/L	4.6 (0.6)	4.5 (0.5)
Baseline eGFR (mL/min per 1.73 m^2^)		
Mean (SD)	43.1 (12.4)	64.4 (24.7)
Distribution		
G1 (>90)	0 (0.0)	2335 (22.7)
G2 (60–89)	454 (10.5)	2854 (27.8)
G3a (45–59)	1328 (30.9)	2378 (23.1)
G3b (30–44)	1898 (44.1)	2062 (20.1)
G4 (15–29)	624 (14.5)	640 (6.2)
G5 (<15)	0 (0.0)	4 (0.0)
Baseline UACR (mg/g)		
Median (IQR)	949 (477–1885)	284 (62–932)
Distribution		
A1 (<30)	1 (0.0)	772 (7.5)
A2 (30–300)	444 (10.3)	4473 (43.5)
A3 (>300)	3859 (89.7)	5028 (48.9)
Medical history		
Baseline T2D mellitus	2906 (67.5)	8875 (86.4)
History of cardiovascular disease	1625 (37.8)	3569 (34.7)
History of heart failure	468 (10.9)	1203 (11.7)
History of stroke	298 (6.9)	767 (7.5)
History of myocardial infarction	392 (9.1)	1760 (17.1)
ACE inhibitor or ARB	4209 (97.8)	9170 (89.3)

Unless otherwise indicated, values for continuous variables are mean (SD); values for categorical variables represent counts (%).

ACE, angiotensin-converting enzyme; ARB, angiotensin II receptor blocker; SD, standard deviation; IQR, interquartile range.

### Time-to-event analysis

To quantify outcomes-based clinical benefit of dapagliflozin beyond the trial periods of DAPA-CKD and DECLARE-TIMI 58, a closed cohort level partitioned survival model was used to predict the outcomes of patients receiving dapagliflozin plus standard therapy versus standard therapy alone. This approach considers the development of a single cohort of patients over time (closed cohort) as opposed to the addition of new, incident-disease patients over time. Here, the partitioned survival approach [15] is used on a clinical event basis to incorporate competing risks of mortality. Patients begin in the event-free partition and, over time, the proportion who die move to a ‘dead’ partition and among the remaining proportion of the cohort, those who are expected to have an event are moved to the ‘event occurred’ partition according to the independently determined survival curves for each clinical event. In subsequent time periods, only patients in the ‘event-free’ partition are at risk of events, so the entire cohort is accounted for across all partitions at every point in time.

The analysis assumes an intention-to-treat perspective and events were considered separately for extrapolation. Non-fatal events were analysed assuming constant hazard in a competing risk framework, with a first-order adjustment for mortality (that is, an adjustment according to general population age/sex mortality). Mortality was taken as the maximum of trial-observed, all-cause mortality (modelled using a generalized gamma distribution) and general population mortality (sex- and age-weighted average, according to top country representation in the trials; Supplementary data, Fig. S1). Only time to first event was considered, thus excluding subsequent events; outcomes were estimated for a cohort size of 1000 patients.

Given the infrequency of some events, a median time (50% of patients) was not expected to be reached within typical patients’ lifetimes; mean times were therefore estimated for consistency. Mean time to first event was calculated as the number of events over the corresponding survival time (defined as time until death of the 1000-patient cohort). For fatal events (all-cause mortality), this time represents mean survival. For non-fatal events, this time frame is the average time until the event occurs in patients who do not die, i.e. patients may die without experiencing a non-fatal event, thus the mean time corresponds only to those who would both experience an event in their lifetime. Uncertainty intervals (95% confidence) were calculated from 1000 bootstrap replicates of the corresponding patient populations.

### Model outcomes

The modelled endpoints were all-cause mortality; kidney failure (sustained eGFR <15 mL/min/1.73 m^2^, initiation of chronic dialysis treatment or receipt of kidney transplant), sustained decline in kidney function (≥40% decline in eGFR compared with baseline) and hospitalization for heart failure. The primary definition of sustained kidney function decline differed between the two trials (DAPA-CKD: ≥50% eGFR decline; DECLARE-TIMI 58: ≥40% eGFR decline); the present analysis was consequently restricted to the ≥40% eGFR decline definition, since only this definition was available consistently across both datasets. The primary outcome for each endpoint was the mean time-to-event in years, with delay defined as the difference in means. Incidence of all outcomes are presented graphically over time, with survival curves plotted for expected incidence over the lifetime of a cohort of 1000 patients.

## RESULTS

### Time-to-event analysis in the DAPA-CKD population

**Figure 2:**
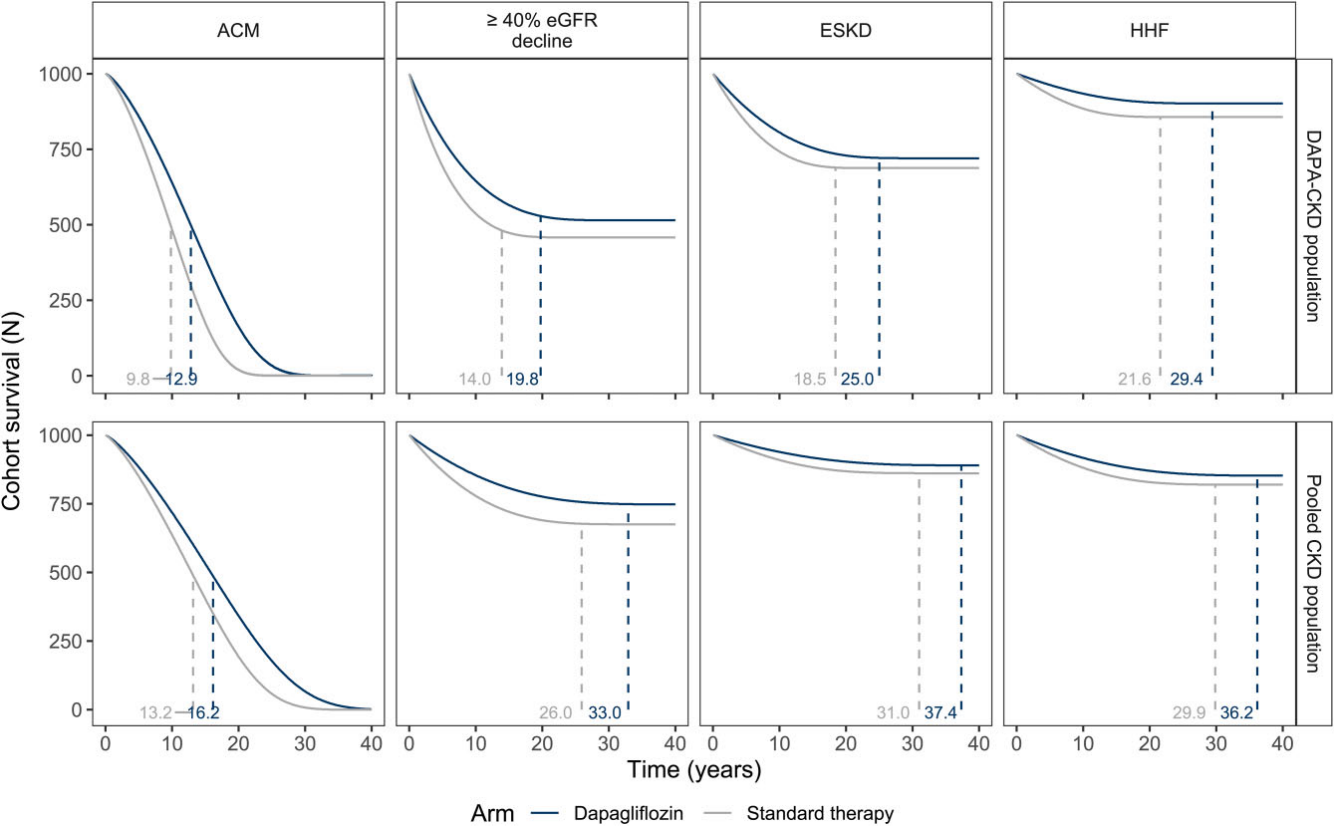
Extrapolated mean time-to-event for ESKD, ≥40% sustained decline in eGFR, hospitalization for heart failure, all-cause mortality for patients treated with dapagliflozin plus standard therapy (blue) vs standard therapy alone. Outcomes are provided per 1000 patients treated with dapagliflozin in addition to standard therapy (blue) versus standard therapy alone (grey). Values indicate the mean time-to-event, conditional on a patient's experiencing an event during the observation period, taken as the predicted lifespan (defined as the first time for survival to reach numerically less than one individual out of the cohort of 1000). ACM: all-cause mortality; ESKD: end-stage kidney disease; HHF: hospitalization for heart failure.

According to modelled estimates, mean overall survival time in the dapagliflozin plus standard therapy arm was estimated to be longer than that of treatment with standard therapy alone (13.1 versus 9.9 years, respectively; delay to death of 3.2 years, Fig. 2).

This modelled survival served as the competing risk for all non-fatal endpoints, as plots depict the time to either the corresponding event or all-cause mortality, whichever comes first (as in clinical trial composite endpoints). As such, the reporting of the mean time to non-fatal events represents a time conditional both on a patient's surviving for the population observation period and experiencing the non-fatal event. In accordance with the DAPA-CKD trial outcome, results of the extrapolated analysis were consistent with an expected delay in CKD progression, as reflected in longer times to sustained decline in kidney function (5.9 years) and kidney failure (6.6 years) for dapagliflozin versus standard therapy alone (Fig. 2, Table 2). Compared with kidney endpoints, there were fewer hospitalization for heart failure events observed in the trial and predicted event rates were correspondingly lower, suggesting a large proportion of patients may not experience such an event (Fig. 2, Table 2). For those that do, the mean time-to-event is predicted to be longer for those treated with dapagliflozin plus standard therapy versus those treated with standard therapy alone.

**Table 2:** Time-to-event (years) for dapagliflozin plus standard therapy versus standard therapy alone.

Endpoint	Dapagliflozin plus standard therapy	Standard therapy	Delay
DAPA-CKD population			
Kidney failure	25.2 (19.3, 31.3)	18.6 (14.8, 23.2)	6.6 (2.8, 10.8)
≥40% eGFR decline	19.8 (15.9, 23.6)	14.0 (11.7, 16.6)	5.9 (3.4, 8.7)
Hospitalization for HF	29.7 (21.7, 38.2)	21.8 (16.9, 28.1)	7.9 (3.1, 13.3)
All-cause mortality	13.1 (10.1, 16.7)	9.9 (8.0, 12.4)	3.2 (0.9, 5.8)
Pooled CKD population			
Kidney failure	35.9 (31.8, 38.2)	29.7 (25.7, 35.2)	6.3 (2.1, 9.5)
≥40% eGFR decline	31.8 (28.4, 33.7)	24.9 (22.0, 28.8)	6.8 (3.8, 9.2)
Hospitalization for HF	34.8 (30.9, 37.0)	28.6 (25.0, 33.7)	6.2 (2.4, 9.2)
All-cause mortality	16.1 (14.1, 18.6)	13.1 (11.8, 14.8)	3.0 (1.5, 4.7)

Values indicate means with 95% confidence intervals in parentheses, derived from 1000 bootstrap replicates of the corresponding trial populations.

HF: heart failure.

### Time-to-event analysis in the pooled CKD population

The pooled CKD population consists of patients with a broader range of CKD, including patients with less severe albuminuria (<200 mg/g) and milder eGFR impairment (>75 mL/min/1.73 m^2^), than the DAPA-CKD population. The pooled CKD population had increased mortality risk factors such as increased age and proportion of T2D in comparison with the DAPA-CKD population (Table 1). Extrapolations of all-cause mortality predicted a delay in mortality (16.1 versus 13.1 years for dapagliflozin plus standard therapy versus standard therapy alone; delay of 3.0 years (Fig. 2, Table 2).

Non-fatal events in the pooled CKD population were subject to the same analysis as for the DAPA-CKD population, and estimates are thus subject to the same conditions of patient survival and experiencing the event. Times to event for non-fatal events were longer for the arm treated with dapagliflozin plus standard therapy versus standard therapy alone, and estimated times for the pooled population were longer than those for corresponding endpoints in the DAPA-CKD population (Fig. 2, Table 2).

The predicted increments in time-to-event differed in magnitude between the analysed populations. Between the DAPA-CKD and pooled CKD analyses, comparable delays were predicted for mean time to kidney failure (6.6 and 6.3 years, respectively), but for decline in kidney function, longer delays were expected in the pooled population (5.9 and 6.8 years, for DAPA-CKD and pooled CKD, respectively). A longer delay in hospitalization for heart failure was estimated in the DAPA-CKD versus pooled CKD analysis (7.9 versus 6.2 years, respectively); however, all-cause mortality was subject to similar delays between the two (3.2 and 3.0 years, DAPA-CKD and pooled CKD, Table 2). A greater proportion of patients in the pooled CKD population was expected to experience hospitalization for heart failure events compared with the DAPA-CKD population (Fig. 2).

## DISCUSSION

The DECLARE-TIMI 58 and DAPA-CKD trials demonstrated substantial benefits to patients with CKD through treatment with dapagliflozin, including reduced risk of kidney failure and hospitalization for heart failure, and lower mortality rates over median follow-up periods of 4.3 and 2.4 years, respectively [6, 9]. In the present analysis, we have extrapolated these outcomes over patients’ lifetimes, suggesting that treatment with dapagliflozin, in addition to standard of care such as angiotensin-converting enzyme inhibitors or angiotensin-receptor blockers, may lead to increased life expectancy and delayed time to adverse cardio-renal outcomes. We estimate that treatment with dapagliflozin may delay progression to kidney failure by an estimated 6.6 years in the DAPA-CKD population—a population considered to be at high risk of progression. A pooled analysis including patients with milder albuminuria and/or eGFR impairment yielded comparable delays in mean time-to-event across renal and cardiovascular events.

This study builds on other extrapolative analyses of SGLT2 inhibitor therapy in patients with CKD using patient-level data from the DAPA-CKD and DECLARE-TIMI 58 trial populations [16, 17]. The delays in deleterious outcomes predicted across the range of CKD disease severity would lead to delayed requirement for dialysis, and/or kidney transplantation. The delay in events is such that a proportion of patients is expected never to reach the event in their lifetime or would reach the event at a later timepoint, with fewer years spent on kidney replacement therapy. Both of these outcomes would lead to an overall reduction in time on dialysis per cohort, thereby reducing the burden of CKD on healthcare resources [4].

From the patient perspective, a delay in reaching kidney failure could substantially improve health related quality of life and additional life years could be spent without the need for kidney replacement therapies, a target relevant to all CKD subpopulations. There is a demonstrated detriment in health-related quality of life for patients initiating dialysis who may already have limited capacity to access these therapies, such as elderly patients; in such cases kidney replacement therapy aimed at increasing life expectancy may not be achievable [18]. In addition, patients who reach kidney failure who are employed are likely to face substantive productivity loss and possibly an inability to work [19]. It is also widely accepted that there is a considerable burden on families and caregivers of patients treated with dialysis [20]; while less quantifiable, a delay in reaching kidney failure could be expected to have a significant impact on the quality of life for caregivers and family groups.

Evidence from clinical trials is crucial to inform on treatment efficacy when the evaluated interventions are to be applied to a wider population in clinical practice. However, the practical limitations of clinical trial design, including focused populations and restricted follow-up, can complicate the estimates of applicability to broader use. Trials typically present outcomes with summary statistics such as hazard ratios, *P*-values and the number needed to treat over the trial follow-up period. However, further characterization of treatment benefits may have a significant impact to patients and healthcare systems beyond a delay of adverse outcomes, which may lead to more patient-centric decision making. Innovative extrapolative methods can supplement traditional statistical analyses to address evidence gaps and broaden understanding of new treatments [21–24].

As with any extrapolative analysis, this analysis is subject to limitations. Any extrapolation beyond observed trial data is subject to uncertainty. However, there is a paucity of long-term evidence to validate the projections, though available evidence may corroborate the evolution of hazards over time, including longer term progression to kidney failure [25], and the impact of age on outcomes [26].

Inherent to parametric survival modelling is the reduction of complex disease processes to a model characterized by only a few parameters. Furthermore, the independent modelling of outcomes meant that risks (of the same or other events) were not modified after event occurrence. The use of such models, however, provides a first estimate of potential future outcomes, and so enables valuable insight to the potential benefits of a treatment. Interpretation of results still requires an element of care, as the extrapolative analysis was aligned to trial inclusion criteria (whether DAPA-CKD or pooled CKD populations).

Pooling available trial data has the advantage of increasing statistical power to observe events and broaden the evidence for use of dapagliflozin across a range of CKD stages, but differences between the trial populations may have influenced the observed results; the DECLARE_CKD_ population trended towards older age and patients with T2D.

In our study, the patient-level data used covered a broad range of CKD severity, but data in those with less severe disease were limited to patients who also had T2D. Patients across a broad range of UACR, with or without T2D, were included in the clinical trial to assess the efficacy and safety of empagliflozin in chronic kidney disease (EMPA-KIDNEY), demonstrating a 28% reduction in the primary composite outcome (progression of kidney disease or death from cardiovascular causes) over a 2.0-year follow-up period [27]. Nevertheless, it did not provide outcomes for constituent components of the composite endpoint stratified by combinations of both mild UACR (<200 mg/g) and mild eGFR impairment (>45 mL/min/1.73 m^2^), thereby obviating direct comparison with the present results. In addition, long-term effects have been previously demonstrated in patients with albuminuric CKD (A3, >300 mg/g) without T2D, where treatment with SGLT2 inhibitors in addition to standard therapy were found to avoid progression to kidney failure [17]. However, our pooled population analysis suggests that SGLT2 inhibitors are also effective in the wider CKD population, including patients with milder disease and low UACR. Future observational studies are needed to validate the clinical outcomes estimated here in a comparable population without T2D.

## CONCLUSION

Our analysis shows that slowing CKD progression through long-term treatment with dapagliflozin may lead to substantial delays in the time to major adverse cardio-renal outcomes such as kidney failure, as well as improving life expectancy. These findings may support informed decision making by patients and clinicians, improving treatment strategies for CKD.

## Supplementary Material

gfae106_Supplemental_File

## Data Availability

Data underlying the findings described in this manuscript may be obtained in accordance with AstraZeneca's data sharing policy described at: https://astrazenecagrouptrials.pharmacm.com/ST/Submission/Disclosure.
