# Supplementary material for: The long-term effects of dapagliflozin in chronic kidney disease: a time-to-event analysis
Source: Nephrol Dial Transplant. 2024 May 10;39(12):2040–7. doi: 10.1093/ndt/gfae106 (PMC11596089; doi:10.1093/ndt/gfae106)
Supplement: gfae106_Supplemental_File [file gfae106_supplemental_file.pdf]

Table of Contents

Supplementary Figures 2

List of Figures

Supplementary Figure S1: Trial versus general population mortality. 2

Supplementary Figures

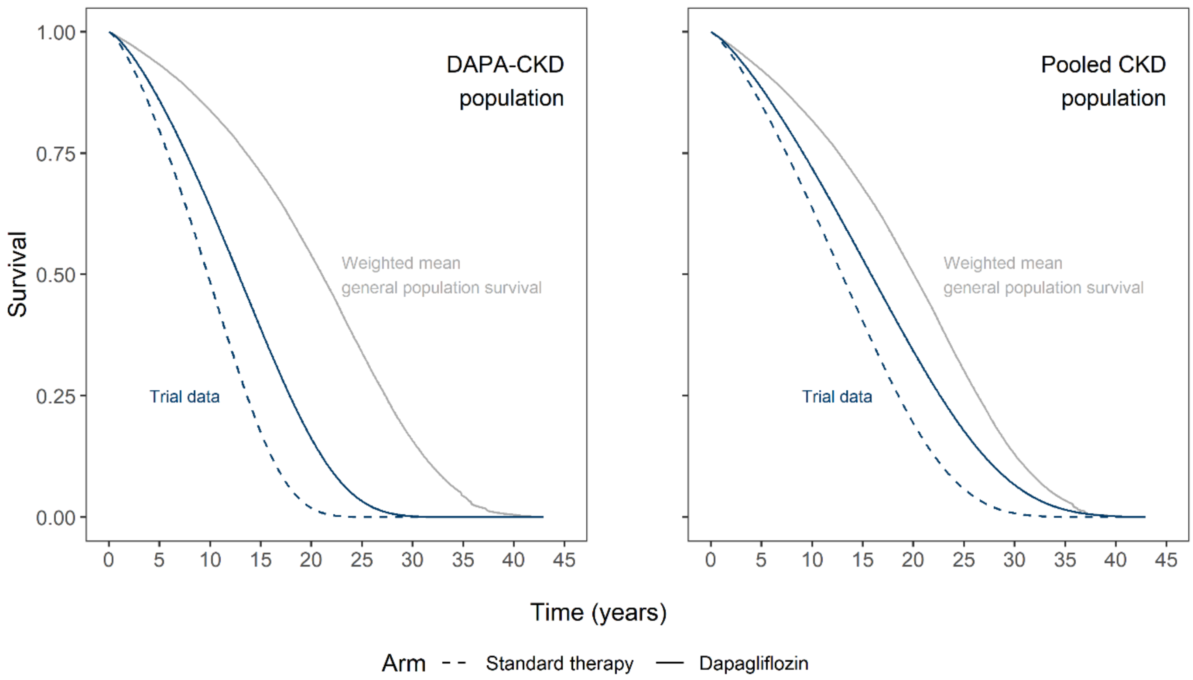

Supplementary Figure S1: Trial versus general population mortality.
All-cause mortality from the DAPA-CKD and the pooled CKD populations were plotted as a function of follow-up, as extrapolated using the generalised gamma distribution. Data are compared with an age- and sex- matched general population average to ensure that the mortality adjustment used in the analysis is not less than the mortality indicated by a comparable general population. CKD: chronic kidney disease
